# Postpartum lumbopelvic pain could be SAPHO syndrome: a case report

**DOI:** 10.3389/fimmu.2025.1614945

**Published:** 2025-08-13

**Authors:** Zheng Weiwei, Zhang Junhong, Zhang Rong

**Affiliations:** ^1^ Department of Geriatrics, Affiliated Hospital of Medical School, Jinling Hospital, Nanjing University, Nanjing, China; ^2^ Nursing Department, Affiliated Hospital of Medical School, Jinling Hospital, Nanjing University, Nanjing, China

**Keywords:** synovitis-acne-pustulosis-hyperostosis-osteitis, SAPHO, lumbopelvic pain, pelvic girdle pain, postpartum women

## Abstract

**Background:**

Synovitis–acne–pustulosis–hyperostosis–osteitis (SAPHO) syndrome is a rare autoimmune disorder. The involvement of spinal and sacroiliac joint in SAPHO syndrome closely resembles the manifestations of postpartum lumbopelvic pain (LPP).

**Case presentation:**

We report a case of a patient whose primary symptom was postpartum LPP, with recurrent episodes lasting 10 months without relief. Unexpectedly, she was diagnosed with SAPHO syndrome in the 11^th^ month when she sought medical attention for severe palmoplantar pustulosis rather than LPP. After combined treatment with Tofacitinib citrate tablets and Etoricoxib tablets, all the pain symptoms disappeared.

**Conclusion:**

For postpartum patients with concurrent palmoplantar scaling and recurrent LPP unresponsive to treatment, clinicians should consider SAPHO syndrome as a potential diagnosis.

## Introduction

Lumbopelvic pain (LPP) is a common problem among postpartum women. It is characterized by lumbar pain [low back pain (LBP], pelvic girdle pain (PGP), or both. Studies indicate that 25% of postpartum women suffer from PGP and/or LBP (1), and 21% of these patients may experience LBP for up to 3 years after delivery (1, 2). Furthermore, 10% of patients have reported disability, reduced quality of life, and diminished capacity for full-time work, for up to 10 years postpartum (3).

Synovitis–acne–pustulosis–hyperostosis–osteitis (SAPHO) syndrome is a rare autoimmune disease (ORPHA:793) which was first described by Chamot et al. in 1987 (4). The estimated incidence of SAPHO syndrome is approximately 0.001% - 0.01%, with 1 to 3 new cases per million population annually (5).However, the actual incidence is difficult to determine, as SAPHO syndrome involves multiple medical specialties. It is characterized by sterile inflammatory osteitis and/or arthritis associated with various dermatological manifestations such as acne, palmoplantar pustulosis, and psoriasis. Osteoarticular involvement most commonly affects the anterior chest wall, followed by the spine, sacroiliac joints, and long bones (6). Patients with SAPHO syndrome are saddled with a heavy disease burden. Yet, the average diagnostic time for the disease is 3.8 ± 5.3 years, which results in significant negative impact on the quality of life of these patients (7).

Lumbopelvic pain (LPP) and SAPHO syndrome cause considerable harm to women. Therefore, there is need for early differential diagnosis, as well as prompt and appropriate treatment for the disease. There are very few studies on pregnancy in relation to SAPHO syndrome; we found only two reported cases of patients who became pregnant after being diagnosed with SAPHO (8, 9).We present the first reported case of a patient whose primary symptom was postpartum LPP characterized by recurrent episodes that were refractory to mitigation or treatment for 10 months. Subsequently, based on concurrent palmoplantar pustulosis, sterile spondylitis, sacroiliitis, and sternoclavicular arthritis, she was diagnosed with SAPHO syndrome. After combined therapy with Tofacitinib citrate and Etoricoxib, her symptoms resolved completely.

## Case presentation

The patient is a nurse in her 30s who has previously had two live births via natural delivery: one in 2020 and the other in 2023. She developed postpartum lower back pain and pelvic girdle pain which persisted for several months without complete relief or cure. Her maternal uncle suffers from ankylosing spondylitis. Additionally, her maternal grandmother has a severe kyphosis, though undiagnosed. The timeline of her disease course is presented in **Table 1**.

**Table 1:** Timeline of the disease in the patient.

Date	Postpartum period (months)	ESR (mm/h)	CRP (mg/L)	Bone/Joint Pain	VAS Score	Palmoplantar scaling	Medication	Treatment effectiveness prior to next visit
Dec 2023	3	33→30	9.76	Lower back/alternating lower limbs	3/2	Mild	Etoricoxib (60mg/day)	Worsened after pain relief
Feb 2024	5	62→38	46.9	Lower back/buttocks/right lower limb	7/8/7	Mild	Etoricoxib (60mg/day) + nerve block therapy (3 sessions).	Worsened after pain relief
Mar 2024	6	17	5.5	Lower back/buttocks/right lower limb/sternoclavicular joint	1/2/1/1	Moderate	Etoricoxib (120mg/day)	Persistent pain despite relief
July 2024	10	25	16.3	Lower back/buttocks/right lower limb/sternoclavicular joint	3/4/2/1	Severe	Etoricoxib (60mg/day) + Tofacitinib citrate tablets (10mg/day)	Significant pain relief/scaling reduced
Aug 2024	11	7	1.2	Lower back/buttocks/right lower limb/sternoclavicular joint	1/1/1/1	Decreased	Etoricoxib (60mg/day) + Tofacitinib citrate tablets (10mg/day)	Pain resolved/scaling absent
Oct 2024	13	13	3.7	Lower back/buttocks/right lower limb/sternoclavicular joint	0/0/0/0	Absent	Etoricoxib (60mg/day) + Tofacitinib citrate tablets (10mg/day)	Not observed

VAS, Visual Analogue Scale.

**Figure 1:**
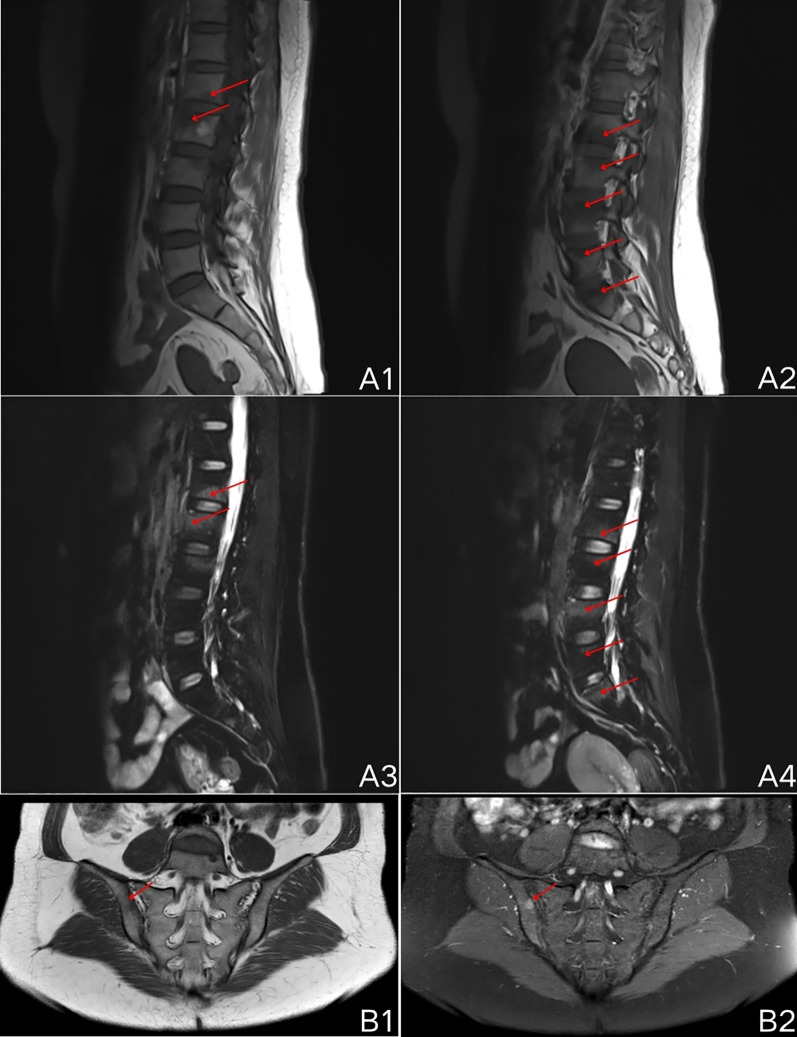
MRI scan results of lumbar spine and sacroiliac joint. **(A1-A4)**, end-plate inflammation (Modic stage I, inflammatory phase) at the opposing margins of L1-L3 vertebrae and the superior margins of L4-S1 vertebrae, T1 sequence shows low signal, T2-FS sequence shows high signal; **(B1, B2)**, abnormal bone signal intensity beneath the right sacroiliac joint surface, T1 sequence shows low signal, PDWI-FS sequence shows high signal.

In December 2023 (3^rd^ month postpartum), the patient was hospitalized in the Spine Surgery Department due to low back pain and alternating bilateral lower limb pain. Admission tests revealed the following results: erythrocyte sedimentation rate (ESR) value of 33 mm/h, and C-reactive protein (CRP) value of 9.76 mg/L. Blood culture showed no bacterial growth, but Next-Generation Sequencing (NGS) revealed *Propionibacterium acnes*. The patient had mild scaling on the palms and soles, which was overlooked. Results from MRI indicated end-plate inflammation at the opposing margins of L1-L3 vertebrae and the upper margins of L4-S1 vertebrae (**Figures 1A1-A4**). The staging of endplate inflammation is Modic stage I (Inflammation phase). She was diagnosed with low back pain and lumbar muscle strain. During hospitalization, her pain was reduced after oral administration of Etoricoxib at a dose of 60mg, given once daily. Pre-discharge tests showed ESR and CRP values of 30 mm/h and 7.0 mg/L, respectively.

In February 2024 (5^th^ month postpartum), the patient was re-admitted in the Spine Surgery Department due to low back pain, hip pain, and persistent right lower limb pain for one month, with poor response to oral Etoricoxib. Admission tests revealed ESR and CRP values of 62 mm/h and 46.9 mg/L, respectively. Blood culture results revealed no bacterial growth. Human leukocyte antigen B27 (HLA-B27) test was negative, and X-ray showed no significant abnormalities in the chest. The patient had mild scaling on the palms and soles, which was overlooked. She was diagnosed with sciatica. During hospitalization, she underwent nerve block therapy three times, with postoperative pain relief. She continued taking oral Etoricoxib after discharge. On February 19, 2024, pre-discharge tests showed ESR value of 38 mm/h.

In March 2024 (6^th^ month postpartum), the patient visited the Spine Surgery Outpatient Department due to sternoclavicular joint pain, lower back pain, hip pain, and right lower limb pain. Sacroiliac joint MRI indicated abnormal bone signal intensity beneath the right sacroiliac joint surface (**Figures 1B1, B2**). The patient exhibited significant scaling on the palms and soles, which was not taken seriously. She was diagnosed with inflammatory spondylopathy (spondyloarthritis), and treatment was continued with oral Etoricoxib (60mg once daily). Four days later, she returned to the outpatient clinic due to persistent nocturnal pain despite Etoricoxib use, and she was advised to increase the dose to 60mg once, twice daily. By April 2024 (7^th^ month postpartum), her pain symptoms had reduced. Tests revealed ESR and CRP values of 17 mm/h and 5.5 mg/L, respectively. She was advised to gradually reduce the dose of Etoricoxib over the next two months.

**Figure 2:**
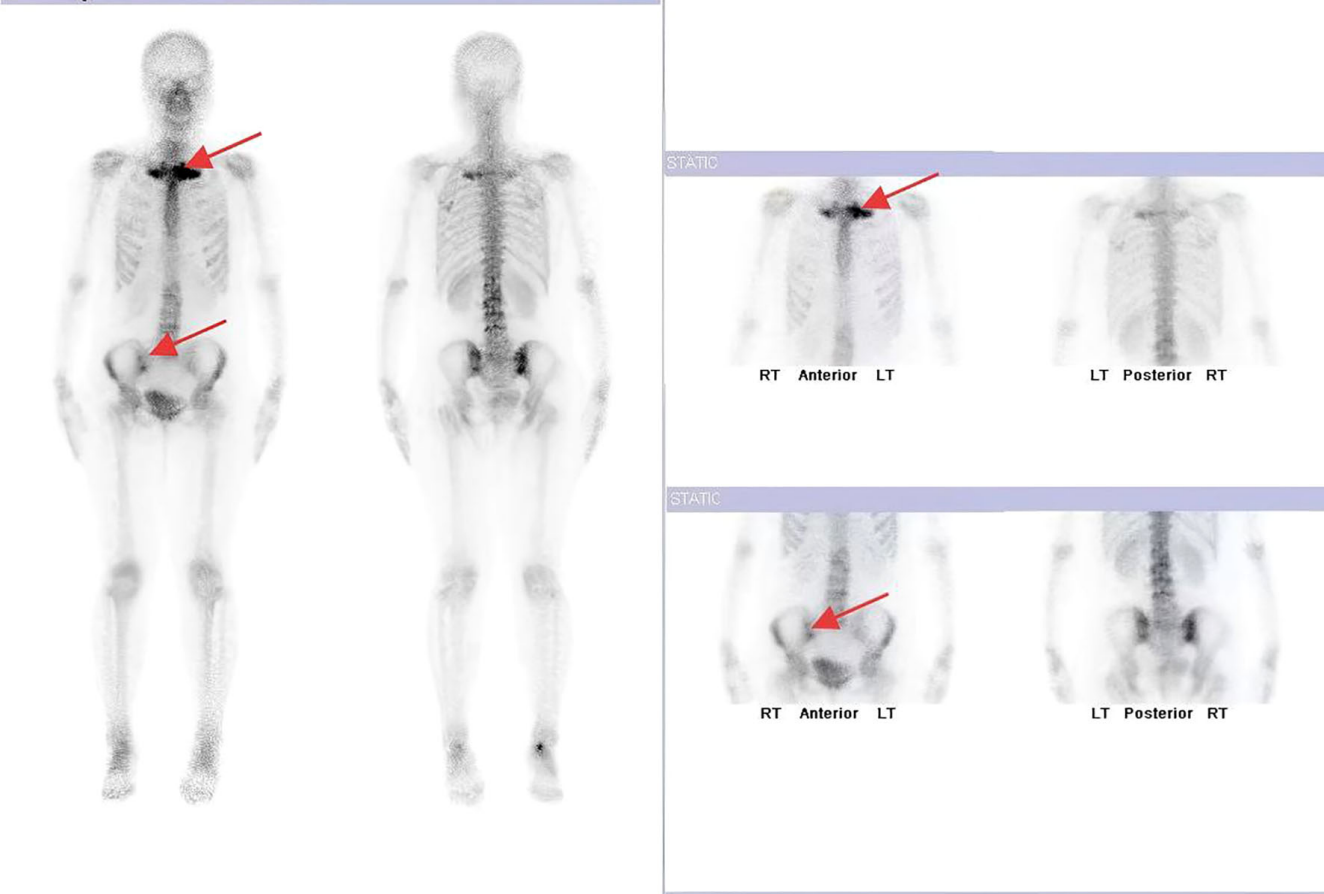
SPECT revealing abnormally increased bone metabolism in the sternoclavicular joints and right sacroiliac joint.

In July 2024 (10^th^ month postpartum), her pain symptoms showed no further reduction, with sternoclavicular joint pain, lower back pain, hip pain, and lower limb pain still persisting. This time around, she visited the Dermatology Department due to severe scaling on the palms and soles. Laboratory tests indicated ESR value of 25 mm/h, and CRP value of 16.3 mg/L. Results from SPECT bone scan revealed inflammation in the sternoclavicular joint and right sacroiliac joint (**Figure 2**). Based on NGS results, laboratory findings, imaging, and symptoms such as lower back pain, sternoclavicular joint pain, and palmoplantar scaling, she was diagnosed with a rare condition, i.e., SAPHO syndrome (10). This diagnosis was hinged on the 2003 diagnostic criteria described by Kahn et al. Several studies have confirmed the efficacy of tofacitinib in alleviating functional impairments associated with SAPHO syndrome (11–14). Therefore, her treatment regimen comprised Tofacitinib citrate tablets (5mg/tablet, twice daily) and a reduced dose of Etoricoxib (60mg/tablet, once daily).

In the 11^th^ month postpartum (August 2024), after continued use of Tofacitinib citrate tablets and Etoricoxib tablets, her pain symptoms were significantly reduced, and the palmoplantar scaling symptoms were alleviated. Laboratory tests indicated ESR and CRP levels of 7 mm/h and 1.2 mg/L, respectively.

**Figure 3:**
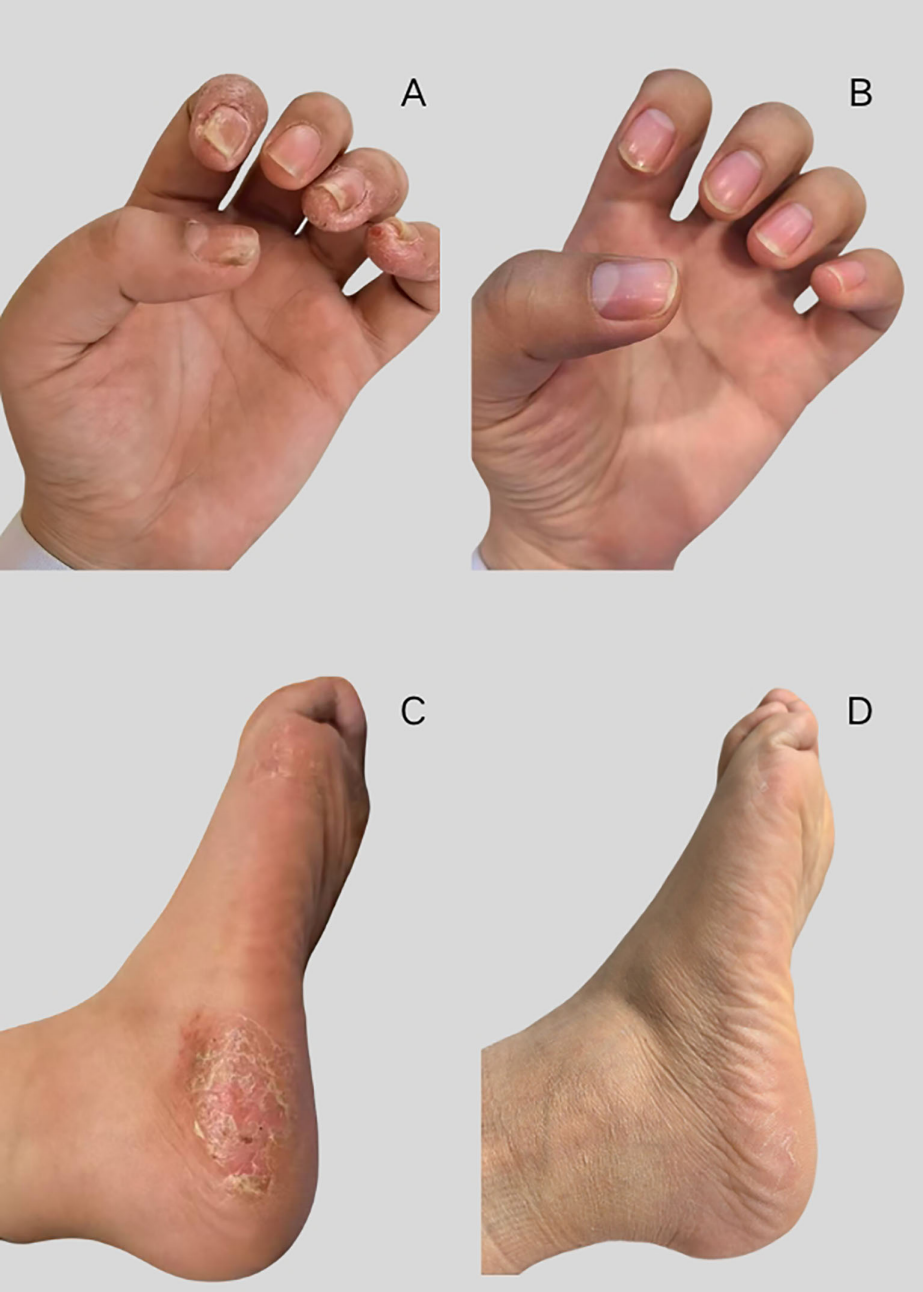
Changes in palmoplantar scaling symptoms. **(A, B)** palmoplantar scaling symptoms, **(C, D)** disappearance of palmoplantar scaling symptoms.

By October 2024 (13^th^ month postpartum), her pain symptoms had resolved, with only mild sternoclavicular joint pain remaining, and palmoplantar scaling symptoms had disappeared. These data are presented in **Figure 3**. Laboratory tests showed ESR value of 13 mm/h and CRP value of 3.7 mg/L. During this examination, the patient was diagnosed with influenza and bronchitis.

**Figure 4:**
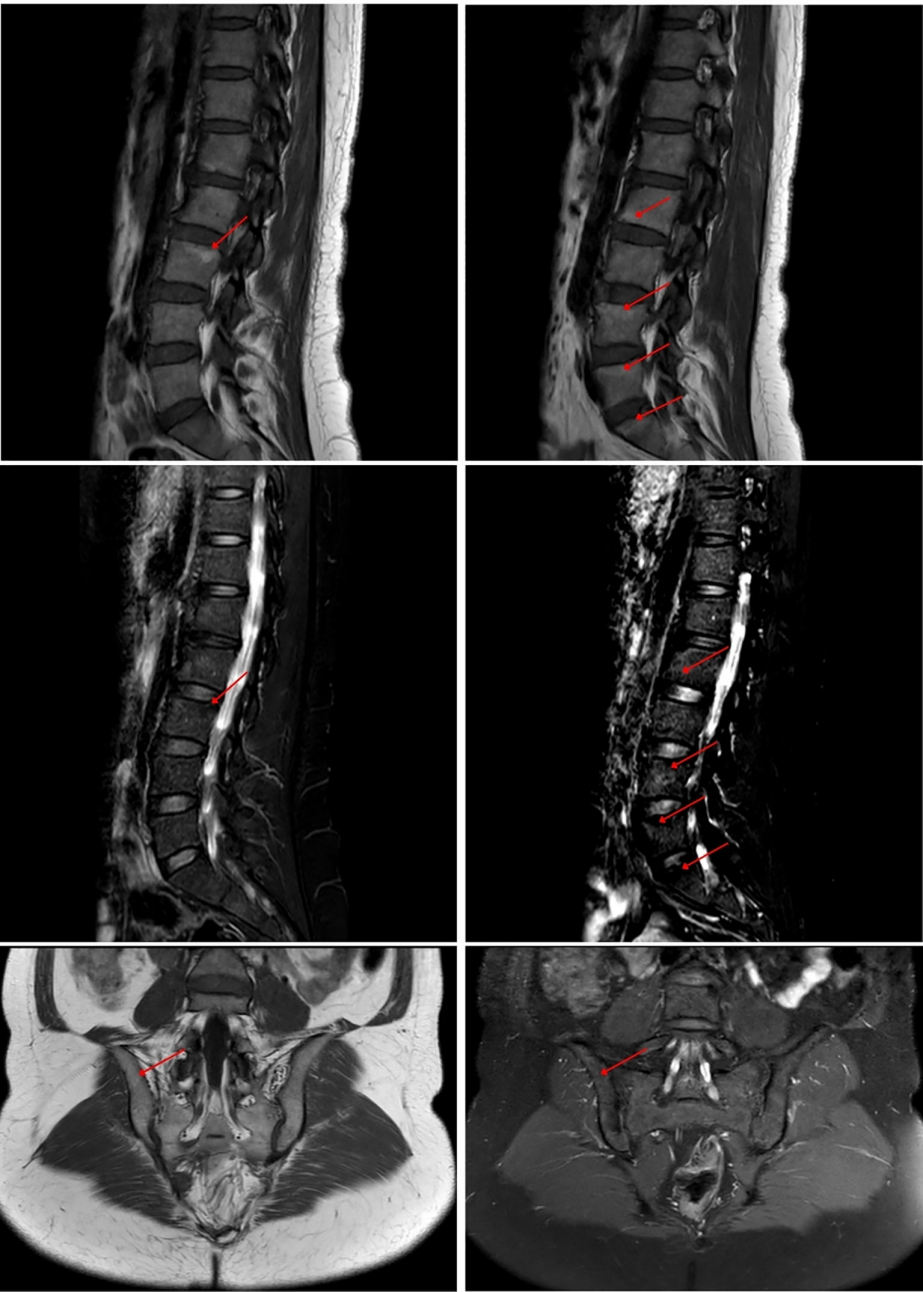
MRI showing end-plate inflammation (Modic stage II, stable phase) at the opposing margins of L2-L3 vertebrae and the superior margins of L4-S1 vertebrae. T1 sequence shows high signal, T2-FS sequence shows low signal.

As of July 2025, all the patient’s pain symptoms and palmoplantar scaling symptoms had essentially disappeared. In July 2025, the latest MRI examination revealed that the edema in the patient’s sacroiliac joint had disappeared. The MRI showed endplate inflammation (Modic stage II, stable phase) on the contralateral edge of the L2-L3 vertebral bodies and the superior edge of the L4-S1 vertebral bodies. The inflammation on the contralateral edge of the L2-L3 vertebral bodies had resolved (**Figure 4**).

## Patient’s perspective

After being diagnosed with SAPHO syndrome, I felt a sense of relief, as if I would finally get better soon. I was certain this was the answer — the illness that had tormented me for so long and hadn’t improved despite so many medications had to be something rare.

## Discussion

This article presents the first reported case of a postpartum patient with chronic lumbopelvic pain and lower limb pain ultimately diagnosed with SAPHO syndrome. Initial examinations by the patient’s physicians included blood cultures and NGS, in order to rule out infectious inflammation. Results from NGS revealed *Propionibacterium acnes* infection. In March 2024, the patient developed palmoplantar pustulosis. However, these manifestations were overlooked in the early stages, thereby delaying the diagnosis.

The etiology of postpartum LPP remains unclear, although mechanical stress on the sacroiliac joints (SI), which is associated with axial spondyloarthritis (SpA), may be a contributing factor. Studies have shown that 59% of women meet ASAS-defined sacroiliitis criteria at 3 months postpartum, and 56% do so at 6 months, while in 41% of women, the criteria persist even at 12 months postpartum (4).

The pathogenesis of SAPHO syndrome remains uncertain. However, it may likely arise from complex interactions among immune dysregulation, genetic predisposition, and environmental factors (15). The pathological features of SAPHO syndrome are axial SpA-like changes affecting sacroiliac joints, spine, and enthesitis, and these symptoms are similar to those of postpartum LPP (16, 17). Approximately 62% of SAPHO patients exhibit spinal/sacroiliac involvement (16, 17), while 94% demonstrate sternoclavicular joint involvement (16). In the case reported here, although sternoclavicular pain was not the initial symptom, it emerged in the 6^th^ postpartum month. Dermatologically, most SAPHO patients (91.9%) present with palmoplantar pustulosis, followed by severe acne (14.3%) and psoriasis vulgaris (15.8%) (18). Notably, cutaneous and osteoarticular manifestations may exhibit asynchronous timing: changes in the skin may be absent, precede, coincide with, or follow joint involvement (15). In the present case report, the patient’s overt palmoplantar pustulosis appeared later than osteoarticular pain.

While there is no current evidence linking pregnancy to SAPHO syndrome, the significant hormonal changes during pregnancy could theoretically trigger the condition, similar to flare-ups seen in other autoimmune diseases (such as systemic lupus erythematosus or multiple sclerosis) (19, 20). Diagnosis remains challenging without specific biomarkers, but SAPHO should be considered when inflammatory arthritis or osteitis (especially in the anterior chest wall, sacroiliac joints, or spine) coexists with psoriasis or neutrophilic/acne-like skin lesions. For diagnosing SAPHO syndrome, the diagnostic criteria proposed by Kahn et al. (5, 10, 21) may serve as a reference, although these criteria have not yet been validated.

Tofacitinib is an inhibitor of JAK1 and JAK3. By inhibiting JAK3, it blocks the expressions of IL-2, IL-4, IL-6, IL-7, IL-9, IL-15, and IL-21, and through JAK1 inhibition, it suppresses IL-6, type 1 interferons, and interferon-γ (22). Several studies have demonstrated the effectiveness of tofacitinib in alleviating the debilitations associated with SAPHO syndrome (11–14). Notably, Li et al. treated 13 SAPHO patients and observed significant reductions in the visual analog scale score for overall osteoarticular pain in the 8^th^ week, as well as marked improvements in the Nail Psoriasis Severity Index score in the 12^th^ week (12). In this case report, the patient showed a significant reduction in joint pain and skin damage after receiving a combination treatment of tofacitinib and etaecoxib for 3 months, which is similar to the treatment duration reported in previous study (12). Slightly different, compared to receiving tofacitinib alone, patients showed a significant reduction in joint pain by the fourth week, while skin damage completely disappeared by the twelfth week.

## Limitations

This study involves only a single patient. Whether pregnancy can trigger SAPHO syndrome, and whether the combination of tofacitinib and etoricoxib is effective in treating postpartum SAPHO, still require long-term follow-up or validation through larger cohort studies.

## Conclusion

Part of the aim of this study was to highlight the diagnostic challenges associated with SAPHO. Clinically, SAPHO syndrome patients with low back pain or sternoclavicular joint pain often present with palmoplantar pustulosis. However, in the early stages, palmoplantar scaling may be subtle or overlooked, thereby complicating accurate diagnosis. Furthermore, if the SAPHO patient is a postpartum woman, the presence of LPP symptoms further obscures the correct diagnosis of SAPHO syndrome.

In summary, this case report reminds us that LPP in postpartum women may not necessarily be pregnancy-related. Therefore, it is advised that, for postpartum patients with concurrent palmoplantar scaling and recurrent LPP unresponsive to treatment, clinicians should consider SAPHO syndrome as a potential diagnosis.

## Data Availability

The original contributions presented in the study are included in the article/supplementary material. Further inquiries can be directed to the corresponding author.
